# Identifying Pathogenic Variants in Vietnamese Children with Functional Single Ventricle Based on Whole-Exome Sequencing

**DOI:** 10.3390/diagnostics15202627

**Published:** 2025-10-17

**Authors:** Le Trong Tu, Nguyen Thi Kim Lien, Nguyen Van Tung, Dang Thi Hai Van, Vu Quynh Nga, Nguyen Tat Tho, Nguyen Thanh Hien, Nguyen Minh Duc, Nguyen Huy Hoang

**Affiliations:** 1 Department of Pediatrics, Hanoi Medical University, Hanoi 100000, Vietnam; trongtu@hmu.edu.vn (L.T.T.); haivan@hmu.edu.vn (D.T.H.V.); nguyentattho1979@gmail.com (N.T.T.); 2 Hanoi Heart Hospital, Hanoi 100000, Vietnam; vuquynhnga@timhanoi.vn; 3 Institute of Biology, Vietnam Academy of Science and Technology, Hanoi 100000, Vietnam; ntkimlienibt@gmail.com (N.T.K.L.); tungnv53@gmail.com (N.V.T.); hienmiu271095.vnu@gmail.com (N.T.H.); 4 Faculty of Biology, Graduate University of Science and Technology, Vietnam Academy of Science and Technology, Hanoi 100000, Vietnam; 5 National Research Center for Medicinal Plant Germplasm and Breeding, National Institute of Medicinal Materials, Hanoi 100000, Vietnam; ducnguyen24vn@gmail.com

**Keywords:** functional single ventricle (FSV), pathogenic variants, phenotype-genotype, whole exome sequencing (WES), Vietnamese patients

## Abstract

**Background:** Functional single ventricle (FSV) comprises a heterogeneous group of congenital heart diseases (CHDs) with severe and complex abnormalities. The multifactorial etiology of the disease poses challenges in identifying specific pathogenic factors and planning effective interventions and preventive treatments for patients. **Methods:** Whole-exome sequencing (WES) was performed to identify variants in relevant genes in 29 FSV patients from different families. **Results:** In total, 95 heterozygous variants across 48 CHD-associated genes were identified, including 85 missense, four small indel, one splicing, one stop gain, and four synonymous variants. Among them, 22 were novels, 11 conflicting, and four pathogenic variants. Each patient carried from two to six variants in different genes, including at least one variant in genes associated with serious heart defects such as *AXIN1*, *BMP2*, *COL6A2*, *GATA4*, *GATA5*, *GDF1*, *MESP1*, *MYH6*, *NFATC1*, *NKX2-6*, *NOTCH1*, *PCSK9*, *TBX1*, *TBX18*, and *TBX20*. In addition, the variants in the *COL6A1*, *CREBBP*, *DOCK6*, *EOGT*, *EP300*, *LRP2*, *MYBPC3*, *MYH7*, *SEMA3C*, and *ZFPM2* genes are associated with characteristic phenotypes of FSV, such as atrial septal defect, ventricular septal defect, small left heart syndrome, transposition of the great arteries, and double outlet right ventricle occurring at high frequency in patients. The prediction results suggest that these are potentially pathogenic variants in patients and may explain the phenotype in patients. **Conclusions:** This is the first study to identify variants associated with functional single ventricle, a complex form of congenital heart disease. Our results contribute to a general understanding of the causes of the disease, thereby guiding treatment and prevention approaches for patients.

## 1. Introduction

The functional single ventricle (FSV) is commonly used to describe any congenital heart defect (CHD) with one functioning ventricle and is the most complex form of CHD. It is uniformly fatal without intervention [1]. FSV is a rare CHD accounting for 1–2% of all CHDs with an incidence of approximately 31 per 100,000 live births [2,3], and 1.6 per 10,000 children living with FSV after infancy [3]. FSV is characterized by abnormal development of the two ventricles: A ventricle is often underdeveloped due to abnormal formation of the ventricles or valves during the embryonic stage [4,5]. FSV is thought to be influenced by both genetic and environmental factors. However, a large proportion of FSV occurs in families with no history of CHD; therefore, it may be due to de novo genetic events, including chromosomal abnormalities, copy number variants, and missense variants [6]. These FSV patients require surgery to repair the defect and ensure basic cardiac function. However, the effectiveness of palliative surgical procedures varies significantly among patients due to differences in the genotype of these patients. Identifying the effects of variants can be useful in management, determining prognosis, and predicting complications; therefore, there is an increasing amount of research aimed at understanding how variations in variants relate to treatment and clinical outcomes in patients [7]. In most patients, the genetic cause of FSV is heterogeneous and polygenic, but like other forms of CHD, it remains poorly understood [6,8,9]. The heterozygous variants have been reported in the *NKX2.5*, *GJA1*, *HAND1*, *TBX5*, *NOTCH1*, and *MYH6* genes in FSV patients [10,11,12]. Studies have shown that the genetic basis of FSV is associated with the overrepresentation of rare variants and de novo copy number variations [13,14,15,16,17]. The FSV phenotype is thought to result from the additive interaction of multiple genes, between genotype and environmental factors, or epigenetic factors. Therefore, evaluating genotype-phenotype correlations to explain the contribution of specific genetic factors to FSV pathogenesis is more difficult [6].

Previous studies have shown overlapping and regulated expression of several genes involved in FSV pathogenesis [18]. Genes encoding important cardiovascular transcription factors such as *NKX2-5*, *GATA4*, *TBX20*, *HAND2*, and *MYOCD* have been identified to be involved in the pathogenesis of CHD [19,20,21,22,23]. Variants in one of the genes of the RAS MAP kinase pathway, including *SOS1*, *RAF1*, *RIT1*, *KRAS*, *SHOC2*, *NRAS*, *SOS2*, *BRAF*, *A2ML1*, *LZTR1*, *MYST4*, *RASA2*, *RRAS*, *SPRY1*, and *SYNGAP1,* may explain an additional 30% of CHD cases [24]. Studies have demonstrated that novel rare variants can affect normal heart development, and common variants can influence important differences in treatment response and clinical outcomes, including survival, remodeling performance, and ventricular function in patients after surgery [25]. Variants associated with increased renin–angiotensin–aldosterone system activity were identified as associated with remodeling and impaired diastolic function after third-stage repair for FSV [26,27]. Lower complication-free survival rates have been reported to be associated with gene variants in adrenergic signaling pathways that increase catecholamine release or sensitivity in FSV patients [28]. Recently, whole-exome sequencing (WES) has greatly assisted in better understanding the genetic basis and improving clinical outcomes.

In this study, we conducted WES to investigate the cause and the correlation between genotype and phenotype in FSV patients to provide some references or suggestions for future genetic diagnosis.

## 2. Materials and Methods

### 2.1. Ethics Statement

The research received ethical approval from the Ethics Committee of the Institute of Genome Research (Approval No. 01-2022/NCHG-HDDD). Written informed consent was obtained from either the participants or their parents/guardians if the participants were minors (<18 years of age). Peripheral blood samples were obtained from the participants after the informed consent forms were signed.

### 2.2. Patients’ Information

Twenty-nine pediatric patients with single ventricle heart disease were recruited from Hanoi Heart Hospital, Vietnam, and used for the study. The patients with a single functional ventricle and/or with a biventricular phenotype but who are not amenable to surgical repair will be selected for the study. Patients with extracardiac malformations and/or known genetic syndromes will be excluded. The patients were aged 1 to 15 years (mean age 4.35 years), including 21 males and eight females. Based on characteristic clinical phenotype [29], the patients were divided into four groups: **(1) Patients with tricuspid atresia (TA)**, atrial septal defects (ASD), ventricular septal defect (VSD), hypoplastic right ventricular, pulmonary valve stenosis (PVS), pulmonary stenosis (PS); transposition of the great arteries (TGA), patent ductus arteriosus (PDA), right ventricular outflow tract obstruction (RVOTO), and double outlet right ventricle (DORV); **(2) Patients with mitral atresia**, hypoplastic left ventricular, DORV, ASD, VSD, TGA, PDA, and atrioventricular discordance; **(3) Patients with DORV**, complete atrioventricular septal defect (CAVSD), PVS, mono-atrial heart, and mono-ventricular; and **(4) Patients with hypoplastic left heart syndrome (HLHS)**, aortic valve stenosis (AS), aortic arch hypoplasia (AAH), bicuspid aortic valve (BAV), ASD, VSD, and PDA (Table 1). Patients underwent peripheral blood sampling for WES to identify the disease-associated variants.

### 2.3. Genetic Analysis

Genomic DNA was extracted using the Qiagen DNA blood mini kit (QIAGEN, Hilden, Germany) according to the manufacturer’s instructions. The DNA samples were measured in concentration on a Nanodrop machine (Thermo Scientific NanoDrop™ 2000, Waltham, MA, USA) and used to construct libraries with the SureSelectXT Reagent Kit (Agilent, Santa Clara, CA, USA). WES was performed using the SureSelect Human All Exon v7 kit (Agilent, CA, USA) on an Illumina sequencer (Illumina, San Diego, CA, USA). The Burrows-Wheeler Aligner (BWA version 0.7.17) was used to align the sequence data to the reference genome (hg19 version 02/2009). Picard (version 2.18.2) and GATK (version 4.0.5.1) tools were used for variant calling and filtering. Variants were annotated using the SnpEff (version 4.3) tool. A list of genes associated with CHD (including 654 genes, Supplementary Table S1) was used to filter pathogenic variants and rare variants with low frequency (MAF < 0.01) (in the SNP database versions 138 and 151). The novel variants were screened from ClinVar (https://www.ncbi.nlm.nih.gov/clinvar/, accessed on 1 September 2025), dbSNP (http://ncbi.nlm.nih.gov/snp/, accessed on 1 September 2025), 1000 Genome Project (http://browser.1000genomes.org, accessed on 1 September 2025), Exome Sequencing Project (https://evs.gs.washington.edu/EVS/, accessed on 1 September 2025), ExAC databases (https://exac.broadinstitute.org, accessed on 1 September 2025), and the in-house database (n = 300). The influence of variants was evaluated with the criteria of the American College of Medical Genetics and Genomics (ACMG) and the in silico prediction tools: CADD (https://cadd.bihealth.org/snv, accessed on 1 September 2025), Fathmm (http://fathmm.biocompute.org.uk/inherited.html, accessed on 1 September 2025), M-CAP (http://bejerano.stanford.edu/mcap/, accessed on 1 September 2025), Mutation Assessor (http://mutationassessor.org/r3/, accessed on 1 September 2025), Mutation Taster (https://www.mutationtaster.org/, accessed on 1 September 2025), PolyPhen2 (http://genetics.bwh.harvard.edu/pph2/, accessed on 1 September 2025), PROVEAN (http://provean.jcvi.org/genome_submit_2.php, accessed on 1 September 2025), SIFT (https://sift.bii.a-star.edu.sg/www/SIFT_seq_submit2.html, accessed on 1 September 2025), and SNP&GO (https://snps-and-go.biocomp.unibo.it/snps-and-go/, accessed on 1 September 2025).

## 3. Results

### 3.1. Phenotype of the Patients in the Study

In this study, we collected and studied WES sequences on 29 pediatric patients with FSV, aged 1 to 15 years (mean age 4.35 years), including 21 males and eight females. Based on phenotypic characteristics, patients were divided into four groups (Table 1).

**Figure 1:**
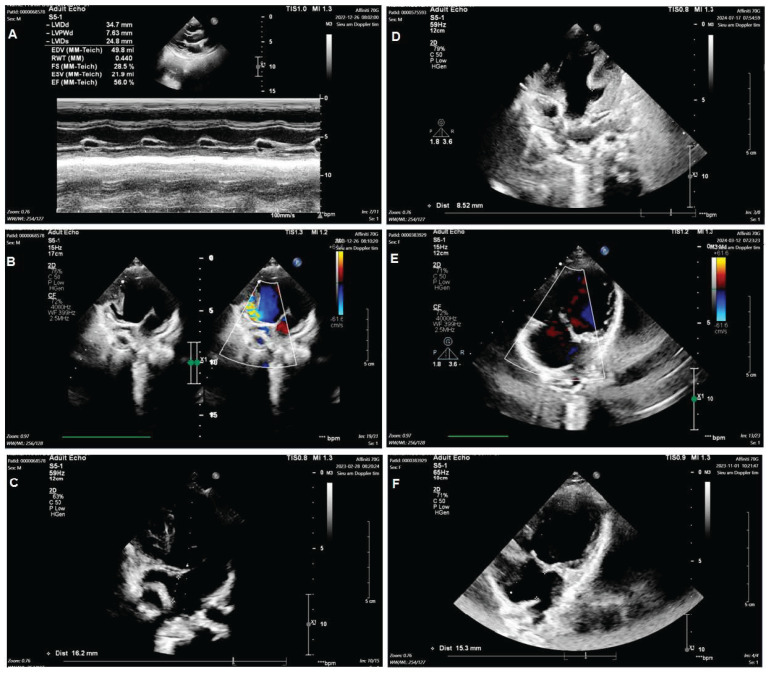
Echocardiogram in the patient with tricuspid valve atresia and in the patient with mitral valve atresia. Including the image of Tricuspid valve atresia (**A**); Ventricular septal defect (**B**); Atrial septal defect (**C**); Mitral valve atresia (**D**); Ventricular septal defect (**E**); Atrial septal defect (**F**) in patient.

**Figure 2:**
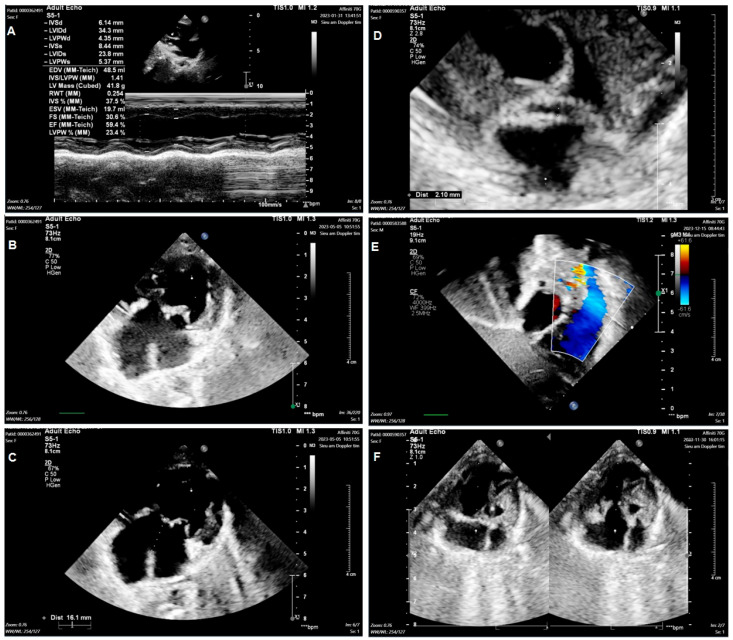
Echocardiogram in the patient with double outlet right ventricle and in the patient with hypoplastic left heart. Including the image of assessment of Ventricular function (**A**); Double outlet right ventricle (**B**); Atrioventricular septal defects (**C**); Hypoplastic left heart (**D**); Pulmonary stenosis (**E**); Aortic stenosis (**F**).

Group 1: Include nine TA patients with clinical features such as ASD, VSD, and PVS/PS (Figure 1A–C). The patients may have hypoplastic right ventricular, right ventricular outflow tract obstruction (RVOTO), DORV, TGA, and PDA. Group 2: Include three mitral valve atresia patients with clinical features such as hypoplastic left ventricular and DORV. The patients may have atrioventricular discordance, PDA, ASD, VSD, and TGA (Figure 1D–F). Group 3: Include twelve DORV patients with clinical features such as CAVSD and PVS/PS (Figure 2A–C). The patients may have a *single atrium* heart, a single ventricle heart, AAH, AS, ASD, VSD, TGA, and a total anomalous pulmonary venous connection (TAPVC). Group 4: Include five HLHS patients with clinical features such as AAH, AS, BAV, ASD, VSD, and PDA (Figure 2D–F).

### 3.2. Genotype of the Patients in the Study

Genetic analysis revealed that 95 potential pathogenic variants (Table 2) in 48 genes associated with CHD (Supplementary Table S2) were identified in the studied patients. Among them, 85 were missense variants, four were small InDel variants, one was a splice variant, one was a stop gain variant, and four were synonymous variants. Twenty-two novel variants, 11 conflicting variants, and three pathogenic variants (c.2107A>C, p.Met703Leu in the *ZFPM2* gene; c.1322C>T, p.Thr441Met in the *MYH7* gene; and c.928G>A, p.Gly310Ser in the *TBX1* gene; according to ClinVar) were identified in these patients. According to the ACMG evaluation criteria, three variants were pathogenic (c.2107A>C, p.Met703Leu in the *ZFPM2* gene; c.1322C>T, p.Thr441Met in the *MYH7* gene; and c.156_157insCCGAGCCCCGT in the *MESP1* gene), two variants were likely pathogenic (c.598C>T, p.Pro200Ser in the *CBS* gene and c.10840G>T, p.Glu3614* in the *TTN* gene), while the remaining variants were evaluated as VUS (Supplementary Table S3). The prediction results showed that all variants were assessed as likely pathogenic and could be causal or explain the phenotype in the patients (Supplementary Table S4). Each patient carried from two to six variants in different genes, one of which was associated with a specific phenotype in each group, such as *AXIN1*, *BMP2*, *COL6A2*, *GATA4*, *GATA5*, *GDF1*, *MESP1*, *MYH6*, *NFATC1*, *NKX2-6*, *NOTCH1*, *PCSK9*, *TBX1*, *TBX18*, and *TBX20*. Patients in Group 1, the tricuspid atresia group, carried variants in the *BMP2*, *MYH6*, *NFATC1*, *NOTCH1*, and *ZFPM2* genes, which have been reported to be associated with tricuspid atresia. Patients in Group 2, the mitral atresia group, carried variants in genes related to mitral atresia, including *EP300*, *NOTCH1*, and *TTN*. Patients in Group 3, the DORV group, carried variants in genes related to DORV, including *GATA4*, *GATA5*, *GDF1*, *NKX2-6*, *NOTCH1*, *SEMA3C*, *TBX1*, *TBX20*, and *ZFPM2*. The analysis data showed that patients also carried variants in genes associated with AVSD (e.g., *COL11A1*, *DNAH11*, *GATA4*, *LRP2*, *MYH6*, *NFATC1*, *NKX2-6*, *NOTCH1*, *SRCAP*, *TBX1*, and *ZFPM2*), TGA (e.g., *COL11A1*, *GDF1*, *LRP2*, *MYH6*, and *TBX1*), or hypoplastic left heart (e.g., *LRP2*, *MYBPC3*, *MYH6*, and *NOTCH1*). Patients in Group 4, the HLHS group, carried variants in genes associated with HLHS, including *KDR*, *MYBPC3*, *MYH6*, and *NOTCH1*. Genes associated with ASD and VSD, such as *MYBPC3* and *NOTCH,* were detected in patients.

## 4. Discussion

In our study, most patients had no family history of CHD or extracardiac anomalies except three patients, P11, P14, and P22, who presented with the heterotaxy syndrome (situs inversus). All patients underwent Fontan surgery to reduce the impact of the defects and improve cardiac function. The patients had phenotypes similar to those described in previous studies [30,31]. FSV represents a spectrum of complex CHDs, often arising from inadequate development of the atrioventricular valves and ventricular chambers. The FSV phenotype can be classified according to the morphology of the dominant ventricle: right ventricle (RV), left ventricle (LV), or unknown, with prevalences of 50–57%, 42–43%, and 0–2%, respectively [32,33,34]. Tricuspid atresia (TA) phenotype (characterized by congenital hypoplasia or agenesis of the tricuspid valve) and mitral atresia phenotype (due to the absence or failure of the valve membrane) are phenotypes described in patients with a small hypoplastic left atrium and a large hypertrophic right atrium. PDA is seen in nearly 80% of patients with FSV [35,36], and PFO or ASD is commonly seen with PFO in two-thirds and ASD in one-third of patients. Patients with FSV typically have a single ventricle. However, in rare cases, patients have two ventricles with a small left ventricle connected to the right ventricle via a small VSD. A large, hypertrophic right ventricle with TGA is the common phenotype compared with the uncommon phenotype of mitral valve atresia with a normal aortic root, a complex form of CHD associated with multiple other defects [30,31].

In the DORV phenotype, the ventricles typically have left ventricular morphology with the outflow tract connecting to the cardiac chambers having right ventricular morphology. The atrioventricular valves may be normal, hypoplastic, stenotic, or atretic. The great arteries are often transposed, with the aorta arising from the hypoplastic right ventricle and the pulmonary artery arising from the LV. PS may be in two-thirds of patients with subvalvular stenosis or valvular pulmonary atresia [1,35]. Valvular and subvalvular stenosis or atresia are in 25–30% while coarctation of the aorta or aortic arch is in nearly 30% of FSV cases [29]. Aortic obstruction is only seen in cases with a normal pulmonary valve in contrast subaortic obstruction can occur in patients with TGA due to VSD stenosis [1,35]. Other lesions such as HLHS, TA, ASD, mitral atresia with normal aortic, and heterotaxy with a single functioning ventricle can be seen in this group.

HLHS is characterized by a very small left ventricle with underdeveloped aortic and mitral valves. The left ventricle is usually a slit-like cavity with thickened muscle, especially when mitral valve atresia is present. The aortic valve is atrophied with hypoplasia of the valve ring. HLHS is the most common (accounting for 25–40%) of all FSVs [3,33]. Other phenotypes such as pulmonary stenosis, pulmonary atresia, disruption of the aortic arch, severe coarctation of the aorta, and occasional hypoplastic aortic arch have also been reported [37,38]. Underdevelopment of the left ventricle, resulting in a single-chamber pumping heart phenotype, the right ventricle, causes this ventricle to work too hard, leading to heart failure that can lead to death or the need for heart transplantation in children with FSV [39]. Several studies have shown that FSV patients with right ventricle morphology have a higher risk of mortality, because the shape, fiber orientation, and metabolic adaptability of the right ventricle are not adapted to support long-term high-pressure systemic circulation [32,40,41].

Previous studies have shown that FSV disease has a relatively high degree of heritability, with a recurrence risk in siblings of 8% [42]. People with a family history of FSV are also at increased risk of developing cardiac defects; in fact, siblings of FSV patients have a 22% higher risk of developping any cardiovascular defect [43]. These data suggest that genetics plays a significant role in the development of FSV. However, Zaidi and Brueckner [6] reported that a large number of CHD cases, especially those with severe malformations, occurred in families with no history of other CHDs. This finding suggests that the cases may be due to de novo variations, including chromosomal abnormalities, small copy number variants, and single-point variants. Exome sequencing studies of participants and their unaffected parents have identified that 10% of CHD is due to de novo variants. If CHD is associated with extracardiac abnormalities, de novo variants could explain 20% of CHD cases [44]. In addition, the genetic information in tailoring care, risk stratification, establishing prognosis, and counseling families affected by CHD is expanding as knowledge of the role of genetics in CHD is gained. The genetic basis of a patient allows for preoperative identification of risk and improved postoperative care for individual patients, despite these patients being physiologically identical [6,25,45].

In Vietnam, patients diagnosed with FSV have undergone surgical correction or palliation for even the most complex FSV, and there has been a remarkable increase in survival for patients with FSV. Although there have been many advances in surgery in hospitals in big cities, medical care and monitoring remain problems when local medical conditions are limited compared to those in big cities in Vietnam. Respiratory complications and myocardial dysfunction are the two most important outcomes after congenital heart surgery that are influenced by genetic background. Therefore, preoperative risk identification enables improved surgical and postoperative care, more tailored to each patient. In addition, elucidating the genetic causes is also important in genetic counseling for patients and their families.

We performed WES to identify variants in genes associated with congenital heart disease that may be the cause of the disease in patients. Genetic analysis showed that the variants identified in these patients were all heterozygous and de novo variants. Each patient carried between two and six variants in different genes, including at least one variant in genes associated with severe heart defects: *AXIN1*, *BMP2*, *COL6A2*, *GATA4*, *GATA5*, *GDF1*, *MESP1*, *MYH6*, *NFATC1*, *NKX2-6*, *NOTCH1*, *PCSK9*, *TBX1*, *TBX18*, and *TBX20* (Table 2). Recent studies have shown that human heart development is tightly controlled and regulated by complex signaling pathways. The key signaling pathways involved include the Nodal/Activin, BMP, WNT, FGF, Notch, Shh, and Vegf signaling pathways. These pathways interact with each other and form signaling networks to regulate and control various processes in the differentiation and development of cells and tissues. In addition, the regulation process has the participation of many related factors, including: Signalling proteins (GDF1, …), Signalling ligands (JAG1, …), Signalling receptors (NOTCH1, NOTCH2, …), Nuclear receptors (NR1D2, NR2F2, …), Transcription factors (CREBBP, NKX2.5, GATA4, GATA6, TBX1, TBX5, TBX20, FOXC1, FOXC2, FOXH1, EP300, NFACT1, ZFPM2, …), Extracellular matrix proteins (ACTC1, ELN, MYH6, MYH7, MYH11, …), … [46,47]. Therefore, variants in the encoding genes for these factors may be the cause leading to the development of an abnormal heart.

**Figure 3:**
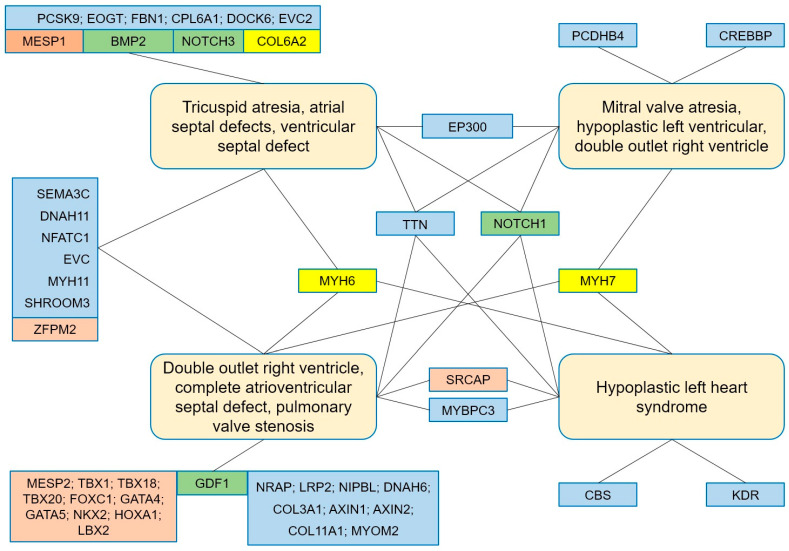

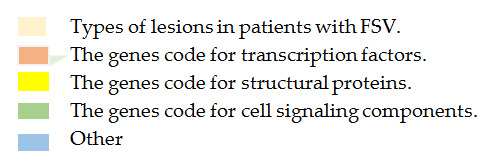
The genetic landscape of lesions in patients with FSV in this study.

**Figure 4:**
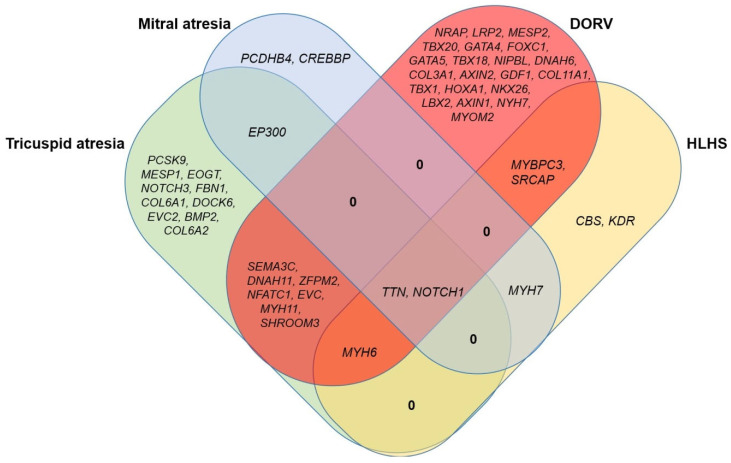
A Venn diagram showing the overlap and unique genes of each group. The *NOTCH1* and *TTN* genes overlap between four groups: Tricuspid atresia, Mitral atresia, DORV, and HLHS. The *MYH6* and *MYH7* genes overlap between three groups: Tricuspid atresia, DORV, and HLHS; and Mitral atresia, DORV, and HLHS, respectively. The *EVC*, *EP300*, *DNAH11*, *MYBPC3*, *MYH11*, *NFATC1*, *SEMA3C*, *SHROOM3*, *SRCAP*, and *ZFPM2* genes overlap between two groups.

In addition, patients in each group carried genetic variants that were specific variants to each group (Figure 3 and Figure 4). Patients in Group 1, the tricuspid atresia group, carried variants in the *BMP2*, *MYH6*, *NFATC1*, *NOTCH1*, and *ZFPM2* genes, which have been reported to be associated with tricuspid atresia [45,48,49,50,51]. Variants in genes associated with atrial septal defects and ventricular septal defects (e.g., *DOCK6*, *EOGT*, *EP300*, *EVC*, *EVC2*, and *SEMA3C*) [6,9] have also been identified in patients. Patients in Group 2, the mitral atresia group, carried variants in the genes including *EP300*, *NOTCH1*, and *TTN* (mitral atresia) [9,49,52], *CREBBP*, *EP300*, and *NOTCH1* (hypoplastic left ventricle) [9,45,49]. Patients in Group 3 carried variants in the genes including *GATA4*, *GATA5*, *GDF1*, *NKX2-6*, *NOTCH1*, *SEMA3C*, *TBX1*, *TBX20*, and *ZFPM2* implicated in DORV [7,45,49]. The analysis data also showed that the patients also carried variants in genes associated with AVSD (e.g., *COL11A1*, *DNAH11*, *GATA4*, *LRP2*, *MYH6*, *NFATC1*, *NKX2-6*, *NOTCH1*, *SRCAP*, *TBX1*, and *ZFPM2*) [7,45,53,54,55], TGA (e.g., *COL11A1*, *GDF1*, *LRP2*, *MYH6*, and *TBX1*) [45,50,53,56], or hypoplastic left heart (e.g., *LRP2*, *MYBPC3*, *MYH6*, *MYH7*, and *NOTCH1*) [45,57,58,59,60]. Patients in Group 4 carried variants in the genes implicated in HLHS including *KDR*, *MYBPC3*, *MYH6*, and *NOTCH1* [45,58,59,61]. Genes associated with ASD and VSD (e.g., *MYH7*, *NOTCH1*, and *SRCAP*) [6,45,62], and BAV (e.g., *CBS*, *MYBPC3*, and *TTN*) [52,58,63] has been detected in patients. Our results showed that most patients had concordance between observed phenotype and determined genotype.

While many genes have been implicated in the pathogenesis of syndromic CHD, identifying genetic variants contributors to non-syndromic CHD is thought to be more difficult due to genetic heterogeneity, incomplete segregation, and potentially oligogenic or polygenic origin [45]. Studies have shown that novel pathogenic variants frequently arise in genes that were highly expressed in the developing heart and several biological pathways such as chromatin remodeling, cilia function, notch signaling, and sarcomere protein-encoding genes are associated with CHD [25]. The involvement of transcription factors in heart development including NKX family members, GATA, T-box, and regulatory factors such as NFATC1 and ZFPM2 form a network responsible for normal cardiac morphogenesis [45]. Transcription factors encoded by the *GATA4* and *GATA5* genes can regulate early cardiac development and play an important role in activating cardiac-specific genes. Variations in these genes lead to severe heart abnormalities in patients with different types of CHD. GATA4 and GATA5 variants have been identified in association with ASD, AVSD, BAV, DORV, PS, TOF, and VSD malformations [6,7,45,49], ASD, AVSD, BAV, DORV, TOF, and VSD [45,49,63], respectively. T-box family genes, including transcription factors important in heart development such as *TBX1* and *TBX20*, have also been reported to be the cause of ASD, AVSD, DORV, LVOTO, TA, TOF, PTA, and VSD [6,7,45,53,64,65]. While variants in the genes encoding regulatory factors NFATC1 and ZFPM2 are responsible for AVSD, DORV, TA, and TOF in patients [45,55,66,67]. In addition, cell signaling and adhesion proteins, encoded by the *NOTCH1* and *GDF1* genes, are thought to play important roles in outflow tract formation and compaction of spongy tissues in cardiac structures [68]. Therefore, any variation in this signaling pathway can cause forms of CHD, in which there is abnormal development of the ventricles [45]. NOTCH1 variants have been identified in patients with severe forms of CHD including AS, ASD, AVSD, BAV, DORV, HLHS, LVOTO, PS, TOF, and VSD [49,55,68].

In addition to the genes regulating heart development mentioned above, genes encoding structural proteins such as MYH6, MYH7, and MYH11 have also been reported to be associated with CHDs [45]. MYH6 variants identified in association with the pathogenesis of ASD [7], AVSD [6], HLHS [59], LVOTO [69], TA, and TGA [50,64] defects in patients. Additionally, HLHS patients at risk of poor outcomes have been associated with *MYH6* variants. Patients carrying *MYH6* variants have abnormal myocardial physiology and reduced right ventricular ejection fraction [58,70]. The transplant-free survival was significantly lower in HLHS patients carrying deleterious variants on MYH6 compared with HLHS patients without the variant [70]. Recently, compound heterozygosity for rare damaging variants in *MYH6* or *MYBPC3*, encoding myosin binding protein C3, was found to be a risk factor for myocardial dysfunction in patients with HLHS [58]. Pathogenic variants in the cardiomyopathy-associated genes, particularly *MYH6*, are associated with both the development of congenital heart defects and impaired myocardial function in these patients [70].

## 5. Limitations

Our study was conducted on patients, so a small number may not fully reflect the relationship between genotype and phenotype in patients. Furthermore, WES alone cannot detect all variants such as chromosomal deletions, duplications, or copy number variations, so it is necessary to combine with methods such as CGHs (Comparative Genomic Hybridization), MLPA (Multiplex Ligation-dependent Probe Amplification)… to identify variations in patients. Although limited, the initial results of our study suggest that genetic variants play a causal role in the disease in patients and emphasize the need for genetic testing and the establishment of a database of disease-associated genetic variants.

## 6. Conclusions

In this study, WES was performed to identify pathogenic variants in 29 patients with FSV from unrelated Vietnamese families. Eighty-five pathogenic variants in 48 genes, including genes associated with severe heart defect *AXIN1*, *BMP2*, *COL6A2*, *GATA4*, *GATA5*, *GDF1*, *MESP1*, *MYH6*, *NFATC1*, *NKX2*-6, *NOTCH1*, *PCSK9*, *TBX1*, *TBX18*, and *TBX20*, were identified. This is the first study to identify variants associated with functional single ventricle, a complex form of congenital heart disease. Our results provide information that contributes to the general understanding of the disease etiology for treatment orientation and to support genetic counseling for patients.

## Figures and Tables

**Table 1:** Phenotypic characteristics of patients.

**Group 1. Tricuspid atresia, atrial septal defects, ventricular septal defect**								
ID	Sex/Age (y)	Hypoplastic Right Ventricular	Pulmonary Valve Stenosis/Pulmonary Stenosis		RVOTO	DORV	Transposition of the Great Arteries	Patent Ductus Arteriosus
P1	F/1		x				x	x
P2	M/6		x		x	x	x	
P3	M/15	x	x		x			
P4	M/8	x	x					x
P5	F/6	x					x	
P6	M/7		x				x	
P7	M/7		x			x		
P8	M/1	x	x					x
P9	M/6	x	x					x
**Group 2. Mitral valve atresia, hypoplastic left ventricular, DORV**								
ID	Sex/Age (y)	VSD	ASD	Transposition of the Great Arteries	Atrioventricular Discordance			Patent Ductus Arteriosus
P10	M/1	x		x	x			x
P11	F/6		x					
P12	M/1			x	x			x
**Group 3.** **Double outlet right ventricle, CAVSD, pulmonary valve stenosis**								
ID	Sex/Age (y)	*Single Atrium*	Single Ventricle	Transposition of the Great Arteries	ASD/VSD	Aortic Valve Stenosis/Aortic Arch Hypoplasia		
P13	F/1		x		x	x		
P14	M/8			x				
P15	M/3	TAPVC		x	x	x		
P16	M/5				x	x		
P17	F/1	x		x	x			
P18	F/5	x		x				
P19	M/1		x	x	x/x			
P20	M/1	TAPVC		x				
P21	F/5		x	x	VSD			
P22	M/15	x	x			x		
P23	M/5			x				
P24	M/4		x	x	x	x		
**Group 4. Hypoplastic left heart syndrome**								
ID	Sex/Age (y)	Aortic Valve Stenosis	Aortic Arch Hypoplasia	BAV	ASD	VSD	Patent Ductus Arteriosus	
P25	M/1	x		**x**	x			
P26	M/1	x	**x**	**x**	x	x	x	
P27	M/1		**x**		**x**	x	x	
P28	M/1	x	**x**		**x**	x	x	
P29	F/1		**x**			x	x	

*ASD, Atrial septal defects; BAV, Bicuspid aortic valve; CAVSD, Complete atrioventricular septal defect; DORV, Double outlet right ventricle; RVOTO, Right ventricular outflow tract obstruction; TAPVC, Total anomalous pulmonary venous connection; VSD, Ventricular septal defect. F, Female; M, Male; y, years*.x, means affected patients.

**Table 2:** Variants identified in patients.

**Group 1. Tricuspid atresia, atrial septal defects, ventricular septal defect**					
**ID/Gene**	**HGVS.c**	**HGVS.p**	**ID/Gene**	**HGVS.c**	**HGVS.p**
**P1**			**P5**		
*BMP2*	c.482T>C rs34183594	p.Leu161Ser	*ZFPM2*	c.2107A>C rs121908603	p.Met703Leu
*EOGT*	c.620+27C>T rs181373917		*MESP1*	c.156_157insCCGAGCCCCGT novel	p.Ala53fs
**P2**			*PCSK9*	c.1026A>G rs509504	p.Gln342Gln
*TTN*	c.103772G>A novel	p.Arg34591Gln	**P6**		
	c.9749T>G rs55634230	p.Val3250Gly	*NOTCH3*	c.4762A>C novel	p.Asn1588His
*EVC*	c.1727G>A rs1383180	p.Arg576Gln	*EP300*	c.6998C>T rs750944383	p.Pro2333Leu
*NOTCH1*	c.2864G>A rs557049479	p.Arg955His	*COL6A1*	c.2662C>T rs368307185	p.Arg888Trp
*MESP1*	c.359T>C rs565846523	p.Leu120Pro	*BMP2*	c.393A>T rs140417301	p.Arg131Ser
*SEMA3C*	c.1009G>A rs1527482	p.Val337Met	**P7**		
**P3**			*MYH6*	c.5410C>A rs144571463	p.Gln1804Lys
*EOGT*	c.1399A>T novel	p.Thr467Ser	*FBN1*	c.1676C>T rs141551765	p.Ala559Val
*DOCK6*	c.6014G>A novel	p.Arg2005His	*COL6A2*	c.185C>T novel	p.Pro62Leu
*COL6A2*	c.3004T>C rs527236952	p.Tyr1002His	**P8**		
*MYH11*	c.5110G>A rs538145374	p.Ala1704Thr	*DNAH11*	c.4306C>T rs183489539	p.Arg1436Trp
*EVC2*	c.2739G>C rs180747811	p.Lys913Asn	*MYH6*	c.5410C>A rs144571463	p.Gln1804Lys
*NFATC1*	c.524G>C rs538981258	p.Ser175Thr	**P9**		
**P4**			*TTN*	c.34216C>A rs532102837	p.Pro11406Thr
*MYH6*	c.5410C>A rs144571463	p.Gln1804Lys	*SHROOM3*	c.2905C>T rs3733245	p.Arg969Trp
*SEMA3C*	c.1009G>A rs1527482	p.Val337Met		c.740A>G rs760622199	p.Asp247Gly
**Group 2. Mitral valve atresia, hypoplastic left ventricular, double outlet right ventricle**					
**ID/Gene**	**HGVS.c**	**HGVS.p**	**ID/Gene**	**HGVS.c**	**HGVS.p**
**P10**			**P12**		
*PCDHB4*	c.599A>G novel	p.Asp200Gly	*TTN*	c.73568C>A rs753557799	p.Pro24523Gln
*NOTCH1*	c.1699A>G rs369067940	p.Ile567Val	*CREBBP*	c.4490A>C novel	p.Lys1497Thr
**P11**				c.4485G>C	p.Lys1495Asn
*TTN*	c.77836G>A novel	p.Ala25946Thr		novel	
*EP300*	c.2971G>C novel	p.Asp991His			
*MYH7*	c.1322C>T rs121913653	p.Thr441Met			
**Group 3. Double outlet right ventricle, complete atrioventricular septal defect, pulmonary valve stenosis**					
**ID/Gene**	**HGVS.c**	**HGVS.p**	**ID/Gene**	**HGVS.c**	**HGVS.p**
*LRP2*	c.233G>C rs546882372	p.Gly78Ala	*TTN*	c.97733A>T novel	p.Asn32578Ile
*NRAP*	c.724C>T rs776357401	p.Pro242Ser	*DNAH11*	c.4306C>T rs183489539	p.Arg1436Trp
*AXIN1*	c.1265G>A rs1187305370	p.Gly422Asp	*NOTCH1*	c.7645C>T rs200893930	p.Arg2549Cys
*AXIN2*	c.1878T>A rs767756290	p.Ser626Arg	**P20**		
**P14**			*MESP2*	c.306C>A rs77473319	p.His102Gln
*FOXC1*	c.1347_1348insAGC novel	p.Gly449_Gly450insSer	*GATA4*	c.790G>A rs201520087	p.Ala264Thr
*ZFPM2*	c.2095C>T novel	p.His699Tyr	*DNAH6*	c.4218T>G rs1398626964	p.Phe1406Leu
**P15**			*COL11A1*	c.2227C>A novel	p.Pro743Thr
*TTN*	c.92336G>C novel	p.Arg30779Thr	**P21**		
	c.46847C>T rs368057764	p.Thr15616Met	*SHROOM3*	c.440T>A rs3821979	p.Leu147His
*NFATC1*	c.2251T>G rs754093	p.Cys751Gly	*DNAH6*	c.4615C>G rs571512486	p.Gln1539Glu
*NRAP*	c.4696C>T rs1885434	p.Arg1566Cys	**P22**		
*SEMA3C*	c.1009G>A rs1527482	p.Val337Met	*TBX18*	c.244_255dupACGTCTGGGCCG	p.Pro85_Ala86insThrSerGlyPro
**P16**			*HOXA1*	c.215_223delATCGCCACC rs544314279	p.His72_His74del
*EVC*	c.1727G>A rs1383180	p.Arg576Gln	*TTN*	c.105876G>A rs372521529	p.Leu35292Leu
*GDF1*	c.985C>T novel	p.Pro329Ser	*COL3A1*	c.3061C>A rs139619440	p.Leu1021Ile
*MYBPC3*	c.2504G>T rs527305885	p.Arg835Leu	*LRP2*	c.6130G>A rs142266106	p.Ala2044Thr
**P17**			**P23**		
*TBX1*	c.928G>A rs41298838	p.Gly310Ser	*NKX2-6*	c.368G>A rs568127693	p.Arg123His
*MYH7*	c.1322C>T rs121913653	p.Thr441Met	*MYH11*	c.5550G>A rs146024732	p.Ser1850Ser
**P18**			*NRAP*	c.4439A>G rs368150420	p.Tyr1480Cys
*TBX20*	c.785C>T novel	p.Thr262Met	*NIPBL*	c.7489G>T rs759115050	p.Val2497Phe
*SRCAP*	c.6397G>A novel	p.Val2133Ile	*SHROOM3*	c.2888C>T rs367782393	p.Ser963Leu
*MYH6*	c.5410C>A rs144571463	p.Gln1804Lys	**P24**		
*LBX2*	c.548C>G rs199798817	p.Pro183Arg	*GATA5*	c.374A>T novel	p.Gln125Leu
*MYOM2*	c.656C>T rs34823600	p.Ala219Val	*NIPBL*	c.3049A>C rs146714879	p.Ile1017Leu
			*SHROOM3*	c.2905C>T rs3733245	p.Arg969Trp
**Group 4. Hypoplastic left heart syndrome**					
**ID/Gene**	**HGVS.c**	**HGVS.p**	**ID/Gene**	**HGVS.c**	**HGVS.p**
**P25**			**P28**		
*TTN*	c.78855T>C rs139953862	p.Asp26285Asp	*MYBPC3*	c.1286C>T rs370412052	p.Ala429Val
*NOTCH1*	c.4864C>T rs747447584	p.Arg1622Cys	*TTN*	c.75137A>C rs759445513	p.Lys25046Thr
**P26**			*MYH6*	c.1262T>C novel	p.Val421Ala
*MYH6*	c.5410C>A rs144571463	p.Gln1804Lys	**P29**		
*CBS*	c.598C>T novel	p.Pro200Ser	*TTN*	c.10840G>T rs540059730	p.Glu3614*
**P27**			*MYH6*	c.428G>A	p.Arg143Gln
*TTN*	c.105642C>A rs560557634	p.Phe35214Leu		rs200377640	
*MYH7*	c.2183C>T rs121913644	p.Ala728Val			
*SRCAP*	c.278A>G rs555507140	p.His93Arg			
*KDR*	c.2524C>T rs41469552	p.Arg842Cys			

Blue genes indicate that they play a major role.

## Data Availability

Data is contained within the article or Supplementary Materials.

## Supplementary Materials

The following supporting information can be downloaded at: https://www.mdpi.com/article/10.3390/diagnostics15202627/s1, Table S1: List of genes (including 654 genes) was used to screen for associated variants. Table S2: Genes identified in the patients. Table S3: Identified variants were evaluated according to ACMG criteria. Table S4: The influence of identified variants using in silico prediction software. References [71,72,73,74,75,76,77,78,79,80,81,82,83,84,85,86,87,88] are cited in the supplementary materials.

## Abbreviations

AAH, Aortic arch hypoplasia; AS, Aortic sterosis; ASD, Atrial septal defect; AV, Atrio-ventricular; AVSDs, AV septal defects; BAV, Bicuspid aortic valve; CAVSD, Complete atrioventricular septal defect; CGH, Comparative genomic hybridization; CHD, Congenital heart disease; DORV, Double outlet right ventricle; FSV, Functional single ventricle; HLHS, Hypoplastic left heart syndrome; LV, Left ventricle; LVOTO, Left ventricular outflow tract obstruction; MLPA, Multiplex ligation-dependent probe amplification; PA, Pulmonary atresia; PDA, Patent ductus arteriosus; PFO, Patent foramen ovale; PS, Pulmonary stenosis; PVS, Pulmonary valve stenosis; RV, Right ventricle; RVOTO, Right ventricular outflow tract obstruction; Severe CHD, Severe congenital heart defects; TA, Tricuspid atresia; TAPVC, Total anomalous pulmonary venous connection; TGA, Transposition of the great arteries; TOF, Tetralogy of Fallot; VSD, Ventricular septal defect; WES, Whole-exome sequencing.
