# Supplementary material for: Identifying Pathogenic Variants in Vietnamese Children with Functional Single Ventricle Based on Whole-Exome Sequencing
Source: Diagnostics (Basel). 2025 Oct 17;15(20):2627. doi: 10.3390/diagnostics15202627 (PMC12564189; doi:10.3390/diagnostics15202627)
Supplement: Supplementary file 1 [file diagnostics-15-02627-s001.zip › Supplementary S1.pdf]

Table S1. List of genes (including 654 genes) was used to screen for associated variants

ABCC6, ABCC9(AD), ABCG5, ACMG, ACT1, ACTA1, ACTA2, ACTB, ACTC(AD), ACTC1(AD), ACTG1, ACTN1, ACTN2(AD), ACVR1, ACVR2B, ADAM17, ADAM19, ADAMTS10, ADAMTSL2, ADK, ADPKD, AHNAK, AK7, AKAP9, AKT3, ALK2, ALPK3, ALX3, AMER1, AMPD1, AMPK, ANK, ANK1, ANK2, ANK3, ANK8, ANKRD1(AD), ANKS6, ANXA11, APC, ARFGEF2, ARHGAP31, ARMC6, ASXL1, ATE1, ATP8, ATRX, ATXN2, AXIN1, AXIN2, B3GALTL, B3GALT6, B3GAT3, BAG3(AD), BAT1, BBS1(AD/AR), BBS2, BCOR, BRAF, BMP2, BMP4, BMP5, BMPR1A (ALK3), BMPR2, BVES, CACNA1, CACNA1C(AD), CACNA1D, CACNA2D1, CACNB2, CACNG4, CALM, CALM1, CALM2, CALM3, CALR2, CALR3, CASQ2, CASZ1, CATI, CAV, CAV3(AD), CBL, CBP, CBS, CCBE1, CCDC11, CCDC40(AR), CCDC141, CD96, CDH2, CDH11, CDKNIC, CDK13, CECR2, CELA1, CEP57, CEP152, CFC1, CHD4, CHD7, CHRD, CHRM2, CHRM3, CHRNA1, CHRND, CHRNG, CHST3, CITED2(AD), CLIP2, CNBP, CNTN1, COL1A1, COL1A2, COL2A1, COL3A1, COL5A1, COL5A2, COL6A1, COL6A2, COL6A3, COL9A1, COL11A1, COL15A1, CORO6, COX7B, COX15, CREBBP, CRELD1(AD), CRKL, CRYAB(AD), CRYPTIC, CSRP3(AD), CSX, CTBP1, CTBP2, CTF1(AD), CTNNA3, CX43, CYFIP1, C5ORF42, DCHS1, DDX11, DES(AD), DHCR2, DHCR7, DHCR24, DIS3L2, DLL3, DLL4, DMD(XR), DMPK, DNAH5, DNAH6, DNAH7, DNAH11(AR), DNAJC19, DND, DOCK6, DOLK(AR), DOT1L, DPMK, DSC2, DSG2(AD), DSG3(AD), DSP(AD), DTNA(AD), DUSP1, DVL1, ECE1, EDEM2, E2F6, EFNB1, EFTUD2, EGFR, EHMT1, ELN, EMB, EMD(XR), ENG, ENPP1, EOGT, EP300, ERK2, ESCO2, ETS1, EVC, EVC2, EYA4(AD), FANCA, FANCC, FANCD2, FBLN, FBLN4, FBLN5, FBN1, FBN2, FGF, FGF8, FGF10, FGFR2, FGFRL1, FHL1, FHL2(AD), FIG4, FKBP12, FKT, FKTN(XR), FLCN, FLNA, FLNB, FLNC(AD), FLT1, FLT4(AD), FOG2, FOXC1/2, FOXD4, FOXH1, FOXL1, FOXP1, FRDA, FRZB, FXN, G6PC3, GAA, GATA3, GATA4(AD), GATA5(AD,AR), GATA6(AD), GATAD1(AR), GATAD4, GBE1, GDF1, GDP1L, GJA1, GJA5, GLA, GLI1, GLI3, GNEB1L, GOLGA4, GPC3, GPC5, GPC6, GPD1L, GTF2I, GTF2IRD1, HADHB, HAND1(AD), HAND2(AD), HANDS, HBB, HCCS, HCN4, HDAC4, HES7, HIRA, HMGCL, HOXA1, HOXD13, HRAS, HRC, HSPB7, HSPG2, HSP90AA1, HYLS1, ID2, IFT43, IFT122, IFT140, IGFBP7, IGFN1, IKBL, ILK(AD), INVS, IQGAP1, IRX4, IRX5, ISL1, ISLET1, ITGA7, ITPR3, JAG1, JAGGED1, JARID2, JPH2(AD), JUN, JUP(AD,AR), KANSL1, KAT6B, KCND3, KCNE1, KCNE2, KCNE3, KCNE4, KCNH2, KCNJ2, KCNJ5, KCNJ8, KCNQ1, KCNQ4, KDM5A, KDM5B, KDM6A, KDR(AD), KIF7, KIF26B, KMT2D, KRAS, LAMA3, LAMA4(AD), LAMB3, LAMP2, LAP2, LBX2, LDB3(AD), LDLR, LEFITY2, LEMD2, LFNG, LIMK1, LIPB1, LMNA(AD), LMNA4(AD), LOX, LRP2, LRRC10, LTPB1, LTPB3, MAML1, MAP1A, MAP2K1, MAP2K2, MAT2A, MATR3, MCTP2, MDM4, MED9, MED12, MED13L, MED15, MEF2, MEF2A, MEF2C, MEGF8, MEIS2(AD), MEGF8, MEIS2, MEK1/2, MESP1, MESP2, MEXN(AD), MFAP5, MGP, MHC2A, MHC6, MIB1, MID1, MKKS, MKL2, MKS1, MLCK, MLC2, MLL2, MLYCD, MMACHC, MMP1, MMP2, MMP13, MMP21(AR), MOC1, MOG1, MPLKIP, MSX1, MTHFR, MTH7, MURC(AD), MYBP2, MYBP3, MYBPC2, MYBPC3(AD), MYCN, MYH2, MYH6(AD), MYH7(AD), MYH7B, MYH8, MYH11, MYLK, MYLK3, MYL1, MYL2(AD), MYL3(AD), MYOCD, MYO18B, MYO19, MYOM1, MYOM2, MYOZ2(AD), MYPN(AD), NAA10, NACA, NBLT, NEBL(AD), NEK1, NEXN(AD), NF1, NFATC1, NFIX, NGR3, NIPA1, NIPA2, NIPBL, NKX2-5(AD), NKX2-6, NNT, NODAL(AD), NOS1, NOS3, NOTCH1(AD), NOTCH2, NOTCHNL, NOTCH3, NPHP3, NPHP4, NPHS1, NRAP, NRAS, NR1D2, NR2F2(AD), NRP1, NSD1, NTRK3, OBSCN(AD), OFD1 (AD), PAFAH1B1, PARP3, PAX6, PCDHB4, PCSK9, PDGF, PDGFRA, PDIA2, PDLIM3, PEX1, PEX5, PIGA, PIGF,

PIGN, PIGT, PIK3CA, PIK3R2, PITX2a, PITX2b, PITX2c, PKD1(AD), PKD2, PKP2(AD), PLB(AD), PLB1, PLEC, PLEKHM2, PLN(AR), PLRG1, PLXND1, PMP22, PPP3CA, PQBP1(XR), PRDM16(AD), PRKAG2, PRKD1, PRKG1, PSEN1(AD), PSEN2(AD), PTCH1, PTCH2, PTPN11(AD), PTPRE, PTPRJ, PTPRM, RAB23, RAD51C, RAF1, RAI1, RBFOX2, RBPJ, RBM2, RBM10, RBM20(AD), RIN4, RIPK4, RIT1, FMR1, RNF20, ROBO1, ROCK2, ROR2, RPS6KA3, RPS19, RSK2, RYR, RYR1, RYR2(AD), SAGD(AD), SALL1, SALL4, SCAP, SCGB, SCN4A, SCN5A(AD), SCN9A, SCN1B, SCN3B, SCN4A, SCN4B, SCN9A, SDHA(AR), SDHD(AD), SEMA3C, SEMA3E, SEMAD6D, SETBP1, SF3B4, SGCD(AD), SHANK3, SHOC2, SHROOM3, SIX1, SIX5, SKI, SKIV2L, SK1, SLC2A10, SLC19A2, SLC22A5, SLC22A20, SLC29A2, SLC29A3, SLC35B2, SMAD1, SMAD2(AD), SMAD3, SMAD4, SMAD6(AD), SMARCA2, SMARCA4, SMC1A, SMC3, SMCHD1, SMOC1, SMOC2, SMURF1, SNAI3, SNIP1, SNTA1, SOD2, SORBS2, SOS1, SOS2, SOX7, SOX9, SPRED1, SRCAP, SREBF1, SRF, STAMBP, STRA6, SYNE2, SYNPO2, TAB2(AD), TAZ(XR), TBL1Y, TBX1(AD), TBX2, TBX3, TBX5(AD), TBX6, TBX18, TBX20(AD), TCAP(AD), TCTN3, TDGF, TDGF1, TECRL, TEP1, TFAP2A, TFAP2B, TGFB2, TGFB3(AD), TGFBK1, TGFBK2, TGFBR1/2, THRAP2, TJP2, TLL1, TMEM43(AD), TMEM237, TMPO(AD), TNNC1(AD), TNNC2(AD), TNNI3(AD,AR), TNNT2(AD), TOP2A, TP63, TPM1(AD), TRDN, TRIM37, TRIM54, TRPC4AP, TRPM4, TSC1, TSC2, TTC37, TTC7A, TTN(AD), TUBAS8, TWIST1, TXNRD2, UBE2B, UBE3B, UBR1, UFD1L, USH2A, USP24, USP44, VCAN, VCL(AD), VEGF, VEGFA, VEGFB, VEGFC, VPS13B, WASHC5, WDPCP, WDR5, WHSC1, WIPI2, WNT3, WNT4, WNT5A, WSTF, XIRP2, YWHAE, ZASP/LDP3, ZBTB17, ZBTB21, ZEB2, ZFPM2, ZIC3, ZMPSTE24, ZNF236, ZNF469, ZNF638, ZNFX1
