# Supplementary material for: Identifying Pathogenic Variants in Vietnamese Children with Functional Single Ventricle Based on Whole-Exome Sequencing
Source: Diagnostics (Basel). 2025 Oct 17;15(20):2627. doi: 10.3390/diagnostics15202627 (PMC12564189; doi:10.3390/diagnostics15202627)
Supplement: Supplementary file 1 [file diagnostics-15-02627-s001.zip › Supplementary S2.pdf]

Table S2. Genes identified in the patients

Gene	OMIM	Phenotype	References
AXIN1	*NM_003502.3*	Severe CHDs	[71]
AXIN2	*NM_004655.3*	BAV	[71]
BMP2	*NM_001200.3*	Severe CHDs, TA	[48]
CBS	*NM_000071.2*	BAV	[63,72]
COL3A1	*NM_000090.3*	BAV	[63]
COL6A1	*NM_001848.2*	AVSD, ASD, BAV, PA, TGA, VSD	[49,63]
COL6A2	*NM_001849.3*	Severe CHDs, AS, AVSD, BAV, PA, PS, TGA	[53,63]
COL11A1	*NM_080629.2*	AVSD, BAV, TGA	[49,63,73]
CREBBP	*NM_004380.2*	AVSD, ASD, HLHS, PDA, CoA, VSD	[49]
DNAH6	*NM_001370.1*	Heterotaxy	[74]
DNAH11	*NM_001277115.1*	AVSD, Heterotaxy	[54]
DOCK6	*NM_020812.3*	ASD, DORV, HLHS, VSD	[9]
EOGT	*NM_001278689.1*	ASD, DORV, HLHS, VSD	[9]
EP300	*NM_001429.3*	ASD, BAV, HLHS, PDA, VSD	[9]
EVC	*NM_153717.2*	AVSD, ASD, VSD	[6]
EVC2	*NM_147127.4*	AVSD, ASD, VSD	[6]
FBN1	*NM_000138.4*	BAV, LVOTO	[75]
FOXC1	*NM_001453.2*	LVOTO, TA, VSD	[66]
GATA4	*NM_001308093.1*	Severe CHDs, ASD, AVSD, BAV, DORV, PS, PVS, TOF, VSD	[6,7,45,49]
GATA5	*NM_080473.4*	Severe CHDs, ASD, BAV, DORV, FSV, LVOTO, TOF, VSD	[45,63]
GDF1	*NM_001492.5*	Heterotaxy, ASD, DORV, LVOTO, TGA, TOF, VSD	[45,76]
HOXA1	*NM_005522.4*	BAV	[77]
KDR	*NM_002253.2*	HLHS	[61]
LBX2	*NM_001282430.1*	ASD	[78]
LRP2	*NM_004525.2*	AVSD, HLHS, PS, TGA	[53,57]
MESP1	*NM_018670.3*	Severe CHDs, DORV, TOF	[79,80]
MESP2	*NM_001039958.1*	DORV	[81]
MYBPC3	*NM_000256.3*	BAV, HLHS	[58,82]
MYH6	*NM_002471.3*	AS, ASD, AVSD, FSV, HLHS, LVOTO, PFO, TA, TGA	[50,55,59,64,69]
MYH7	*NM_000257.3*	ASD, FSV, HRHS, VSD	[6,60]
MYH11	*NM_001040114.1*	BAV, PDA	[6,45,63,64]
MYOM2	*NM_003970.3*	TOF	[83]
NFATC1	*NM_001278675.1*	Severe CHDs, AVSD, TA	[45,66,67]
NIPBL	*NM_133433.3*	ASD, AVSD, PDA, PVS, VSD	[9,84]
NKX2-6	*NM_001136271.2*	Severe CHDs, AVSD, DORV, PDA, TOF, TA	[6,7]
NOTCH1	*NM_017617.4*	Severe CHDs, AS, ASD, AVSD, BAV, DORV, HLHS, LVOTO, PS, TA, TOF, VSD	[45,49,55,68]
NOTCH3	*NM_000435.2*	BAV	[68]
NRAP	*NM_001261463.1*	FSV	[85]
PCDHB4	*NM_018938.3*	TOF	[56]
PCSK9	*NM_174936.3*	Severe CHDs	[86]

SEMA3C	*NM_006379.3*	ASD, DORV, TOF, VSD	[6,49]
SHROOM3	*NM_020859.3*	Heterotaxy	[87]
SRCAP	*NM_006662.2*	ASD, AVSD	[62]
TBX1	*NM_080647.1*	Severe CHDs, ASD, AVSD, DORV, PS, TA, TGA, TOF, VSD	[45,53,64,65]
TBX18	*NM_001080508.2*	Severe CHDs	[88]
TBX20	*NM_001077653.2*	Severe CHDs, AS, ASD, FSV, DORV, LVOTO, PDA, PFO, TOF, VSD	[6,7]
TTN	*NM_001267550.2*	BAV	[45,52,55]
ZFPM2	*NM_012082.3*	AVSD, DORV, TA, TOF	[51]

Aortic sterosis (AS); Atrial septal defect (ASD); Atriventricular septal defect (AVSD); Bicuspid aortic valve (BAV); Functional single ventricular (FSV); Hypoplastic left heart syndrome (HLHS); Hypoplastic right heart syndrome (HRHS); Left ventricular outflow tract obstruction (LVOTO); Pulmonary atresia (PA); Patent ductus arteriosus (PDA); Patent foramen ovale (PFO); Pulmonary stenosis (PS); Pulmonary valve stenosis (PVS); Severe congenital heart defects (severe CHDs); Tricuspid atresia (TA); Total anomalous pulmonary venous connection (TAPVC); Transposition of the great arteries (TGA); Tetralogy of Fallot (TOF); Ventricular septal defect (VSD)
