# Supplementary material for: Identifying Pathogenic Variants in Vietnamese Children with Functional Single Ventricle Based on Whole-Exome Sequencing
Source: Diagnostics (Basel). 2025 Oct 17;15(20):2627. doi: 10.3390/diagnostics15202627 (PMC12564189; doi:10.3390/diagnostics15202627)
Supplement: Supplementary file 1 [file diagnostics-15-02627-s001.zip › Supplementary S3.pdf]

Table S3. Identified variants were evaluated according to ACMG criteria

ID/Gene	Chr	Start	HGVS.c	HGVS.p	ACMG	Evidence
**P1**						
BMP2	20	6,759,027	c.482T>C	p.Leu161Ser	VUS	PM2_Supporting
EOGT	3	69,053,502	c.620+27C>T		VUS	PM2_Supporting
**P2**						
TTN	2	179,397,570	c.103772G>A	p.Arg34591Gln	VUS	PM2_Supporting
	2	179,629,493	c.9749T>G	p.Val3250Gly	VUS	PM2_Supporting<br>PP3
EVC	4	5,785,442	c.1727G>A	p.Arg576Gln	Conflicting	
NOTCH1	9	139,404,290	c.2864G>A	p.Arg955His	VUS	PP2
MESP1	15	90,294,104	c.359T>C	p.Leu120Pro	VUS	PM2_Supporting<br>PP3
SEMA3C	7	80,427,530	c.1009G>A	p.Val337Met	B	BP6
**P3**						
EOGT	3	69,027,522	c.1399A>T	p.Thr467Ser	VUS	PM2_Supporting
DOCK6	19	11,311,071	c.6014G>A	p.Arg2005His	VUS	PM2_Supporting
COL6A2	21	47,552,410	c.3004T>C	p.Tyr1002His	VUS	PM2_Supporting<br>PP3
MYH11	16	15,813,159	c.5110G>A	p.Ala1704Thr	VUS	PM2_Supporting
EVC2	4	5,617,239	c.2739G>C	p.Lys913Asn	Conflicting	
NFATC1	18	77,170,799	c.524G>C	p.Ser175Thr	VUS	PM2_Supporting
**P4**						
MYH6	14	23,853,806	c.5410C>A	p.Gln1804Lys	VUS	PM2_Supporting<br>PP3
SEMA3C	7	80,427,530	c.1009G>A	p.Val337Met	B	BP6
**P5**						
ZFPM2	8	106,814,417	c.2107A>C	p.Met703Leu	P	PM1,<br>PM2_Supporting
MESP1	15	90,294,306	c.156_157insCC<br>GAGCCCCGT	p.Ala53fs	P	PVS1<br>PM5_Supporting
PCSK9	1	55,523,033	c.1026A>G	p.Gln342Gln	Conflicting	
**P6**						
NOTCH3	19	15,281,611	c.4762A>C	p.Asn1588His	VUS	PM5_Supporting<br>PP3
EP300	22	41,574,713	c.6998C>T	p.Pro2333Leu	VUS	PP3
COL6A1	21	47,423,502	c.2662C>T	p.Arg888Trp	VUS	PM2_Supporting
BMP2	6	6,758,938	c.393A>T	p.Arg131Ser	VUS	PM2_Supporting<br>PP3
**P7**						
MYH6	14	23,853,806	c.5410C>A	p.Gln1804Lys	VUS	PM2_Supporting<br>PP3
FBN1	16	48,802,279	c.1676C>T	p.Ala559Val	VUS	PM1, PP2
COL6A2	21	47,531,962	c.185C>T	p.Pro62Leu	VUS	PM2_Supporting<br>PP3
**P8**						
DNAH11	7	21,658,769	c.4306C>T	p.Arg1436Trp	VUS	PM2_Supporting
MYH6	14	23,853,806	c.5410C>A	p.Gln1804Lys	VUS	PM2_Supporting<br>PP3
**P9**						
TTN	2	179,542,423	c.34216C>A	p.Pro11406Thr	VUS	PM2_Supporting
SHROOM3	4	77,662,231	c.2905C>T	p.Arg969Trp	VUS	PM2_Supporting<br>PP3

	4	77,660,066	c.740A>G	p.Asp247Gly	VUS	PM2_Supporting
**P10**						
PCDHB4	5	140,502,179	c.599A>G	p.Asp200Gly	VUS	PM5
NOTCH1	9	139,410,139	c.1699A>G	p.Ile567Val	VUS	PM2_Supporting
**P11**						
TTN	2	179,433,023	c.77836G>A	p.Ala25946Thr	VUS	PM5
EP300	22	41,547,990	c.2971G>C	p.Asp991His	VUS	PM5
MYH7	20	23,898,249	c.1322C>T	p.Thr441Met	P	PM1
**P12**						
TTN	2	179,437,291	c.73568C>A	p.Pro24523Gln	VUS	PP3
CREBBP	16	3,786,721	c.4490A>C	p.Lys1497Thr	VUS	PM5, PP3
	16	3,786,726	c.4485G>C	p.Lys1495Asn	VUS	PM5, PP3
**P13**						
LRP2	2	170,175,349	c.233G>C	p.Gly78Ala	VUS	PM5
NRAP	10	115,410,256	c.724C>T	p.Pro242Ser	VUS	PP3
AXIN1	16	348,241	c.1265G>A	p.Gly422Asp	VUS	PP3
AXIN2	17	63,533,016	c.1878T>A	p.Ser626Arg	VUS	PP3
**P14**						
FOXC1	6	1,612,025	c.1347_1348insAGC	p.Gly449_Gly450insSer	VUS	PM2_Supporting
ZFPM2	8	106,814,405	c.2095C>T	p.His699Tyr	VUS	PM2_Supporting
**P15**						
TTN	2	179,414,017	c.92336G>C	p.Arg30779Thr	VUS	PM2_Supporting
	2	179,483,430	c.46847C>T	p.Thr15616Met	VUS	PM2_Supporting
NFATC1	18	77,246,406	c.2251T>G	p.Cys751Gly	VUS	PP3
NRAP	10	115,350,597	c.4696C>T	p.Arg1566Cys	VUS	PP3
SEMA3C	7	80,427,530	c.1009G>A	p.Val337Met	B	BP6
**P16**						
EVC	4	5,785,442	c.1727G>A	p.Arg576Gln	Conflicting	
GDF1	19	18,979,540	c.985C>T	p.Pro329Ser	VUS	PM2_Supporting
MYBPC3	11	47,359,040	c.2504G>T	p.Arg835Leu	VUS	PM2_Supporting
**P17**						
TBX1	22	19,753,444	c.928G>A	p.Gly310Ser	B	BP6
MYH7	14	23,898,249	c.1322C>T	p.Thr441Met	VUS	PP2
**P18**						
TBX20	7	35,280,519	c.785C>T	p.Thr262Met	VUS	PM2_Supporting PP3
SRCAP	16	30,745,022	c.6397G>A	p.Val2133Ile	VUS	PM2_Supporting PP3
MYH6	14	23,853,806	c.5410C>A	p.Gln1804Lys	VUS	PM2_Supporting PP3
LBX2	2	74,725,103	c.548C>G	p.Pro183Arg	VUS	PP2_Supporting
MYOM2	8	2,017,399	c.656C>T	p.Ala219Val	VUS	PP3
**P19**						
TTN	2	179,406,071	c.97733A>T	p.Asn32578Ile	VUS	PM2_Supporting
DNAH11	7	21,658,769	c.4306C>T	p.Arg1436Trp	VUS	PM2_Supporting
NOTCH1	9	139,390,546	c.7645C>T	p.Arg2549Cys	VUS	PP2_Supporting
**P20**						
MESP2	15	90,319,894	c.306C>A	p.His102Gln	VUS	PM2_Supporting
GATA4	8	11,607,623	c.790G>A	p.Ala264Thr	VUS	PM2_Supporting
DNAH6	2	84,851,609	c.4218T>G	p.Phe1406Leu	VUS	PM2_Supporting PP2
COL11A1	1	103,463,871	c.2227C>A	p.Pro743Thr	VUS	PM2_Supporting PP3

**P21**						
SHROOM3	4	77,631,425	c.440T>A	p.Leu147His	VUS	PP3
DNAH6	2	84,861,727	c.4615C>G	p.Gln1539Glu	VUS	PM2_Supporting PP2
**P22**						
TBX18	6	85,473,644	c.244_255dupACGTCTGGGCCG	p.Pro85_Ala86insThrSerGlyPro	VUS	PM5, PM4
HOXA1	7	27,135,308	c.215_223delATCGCCACC	p.His72_His74del	VUS	PM5, PM4
TTN	2	179,395,466	c.105876G>A	p.Leu35292Leu	VUS	PM2_Supporting
COL3A1	2	189,870,953	c.3061C>A	p.Leu1021Ile	VUS	PM2_Supporting
LRP2	2	170,068,628	c.6130G>A	p.Ala2044Thr	VUS	PM2_Supporting PP3
**P23**						
NKX2-6	8	23,560,502	c.368G>A	p.Arg123His	VUS	PM2_Supporting
MYH11	16	15,809,105	c.5550G>A	p.Ser1850Ser	VUS	PM2_Supporting
NRAP	10	115,355,479	c.4439A>G	p.Tyr1480Cys	VUS	PM2_Supporting PP3
NIPBL	5	37,059,071	c.7489G>T	p.Val2497Phe	VUS	PM2_Supporting PP2
SHROOM3	4	77,662,214	c.2888C>T	p.Ser963Leu	VUS	PM2_Supporting
**P24**						
GATA5	20	61,050,204	c.374A>T	p.Gln125Leu	VUS	PM2_Supporting PP3
NIPBL	5	36,986,331	c.3049A>C	p.Ile1017Leu	VUS	PM2_Supporting PP2
SHROOM3	4	77,662,231	c.2905C>T	p.Arg969Trp	VUS	PM2_Supporting
**P25**						
TTN	2	179,432,004	c.78855T>C	p.Asp26285Asp	VUS	PM2_Supporting
NOTCH1	9	139,399,279	c.4864C>T	p.Arg1622Cys	VUS	PM2_Supporting PP2
**P26**						
MYH6	14	23,853,806	c.5410C>A	p.Gln1804Lys	VUS	PM2_Supporting PP3
CBS	21	44,485,565	c.598C>T	p.Pro200Ser	LP	PM5, PP2
**P27**						
TTN	2	179,395,700	c.105642C>A	p.Phe35214Leu	VUS	PM2_Supporting
MYH7	14	23,895,007	c.2183C>T	p.Ala728Val	VUS	PM2_Supporting
SRCAP	16	30,715,608	c.278A>G	p.His93Arg	VUS	PM2_Supporting
KDR	4	55,963,919	c.2524C>T	p.Arg842Cys	VUS	PP3
**P28**						
MYBPC3	11	47,364,637	c.1286C>T	p.Ala429Val	VUS	PM2_Supporting
TTN	2	179,435,722	c.75137A>C	p.Lys25046Thr	VUS	PM2_Supporting
MYH6	14	23,870,066	c.1262T>C	p.Val421Ala	VUS	PM2_Supporting
**P29**						
TTN	2	179,621,363	c.10840G>T	p.Glu3614*	LP	PVS1, PM2_Supporting
MYH6	14	23,874,506	c.428G>A	p.Arg143Gln	VUS	PM2_Supporting PP3

Chr: Chromosome; LP: Likely pathogenic; P: Pathogenic; VUS: Variants of uncertain significance.
