# Supplementary material for: Identifying Pathogenic Variants in Vietnamese Children with Functional Single Ventricle Based on Whole-Exome Sequencing
Source: Diagnostics (Basel). 2025 Oct 17;15(20):2627. doi: 10.3390/diagnostics15202627 (PMC12564189; doi:10.3390/diagnostics15202627)
Supplement: Supplementary file 1 [file diagnostics-15-02627-s001.zip › Supplementary S4.pdf]

Table S4. The influence of identified variants using in silico prediction software

ID/Gene	**HGVS.c / HGVS.p**	dbSNP/MAF	CADD	Fathmm MKL	Mutation assess	Mutation taster	SIFT	PolyPhen	PROVEAN	SNP& GO	MCAP
**P1**	**Group 1. Tricuspid atresia**										
BMP2	c.482T>C<br>(p.Leu161Ser)	rs34183594<br>0.000199681	34.00<br>D	0.800<br>D	-	Benign	0.457<br>T	0.780<br>B	-	RI8<br>Disease	-
EOGT	c.620+27C>T	rs181373917<br>0.00199681					-				
**P2**											
TTN	c.103772G>A<br>(p.Arg34591Gln)	novel	24.20<br>D	0.955<br>D	-	Benign	0.000<br>D	1.000<br>D	-	-	-
	c.9749T>G<br>(p.Val3250Gly)	rs55634230	22.90<br>D	0.993<br>D	-	Benign	0.066<br>D	0.987<br>D	-	-	-
EVC	c.1727G>A<br>(p.Arg576Gln)	rs1383180					conflicting				
NOTCH1	c.2864G>A<br>(p.Arg955His)	rs557049479<br>0.000599042					conflicting				
MESP1	c.359T>C<br>(p.Leu120Pro)	rs565846523	24.60<br>D	0.986<br>D	-	Delete-<br>rious	0.000<br>D	1.000<br>D	-	RI4<br>Disease	-
SEMA3C	c.1009G>A<br>(p.Val337Met)	rs1527482<br>0.0399361	32.00<br>D	0.983<br>D	-	Benign	0.000<br>D	0.998<br>D	-	RI1<br>Neutral	-
**P3**											
EOGT	c.1399A>T<br>(p.Thr467Ser)	novel	24.60<br>D	0.996<br>D	-	Delete-<br>rious	0.046<br>D	0.993<br>D	-	RI2<br>Neutral	-
DOCK6	c.6014G>A<br>(p.Arg2005His)	novel	28.30<br>D	0.986<br>D	-	Delete-<br>rious	0.002<br>D	1.000<br>D	-	RI8<br>Neutral	-
COL6A2	c.3004T>C<br>(p.Tyr1002His)	rs527236952<br>0,000199681	23.10<br>D	0.944<br>D	-	Benign	0.000<br>D	0.930<br>D	-	RI9<br>Neutral	-
MYH11	c.5110G>A<br>(p.Ala1704Thr)	rs538145374<br>0,000399361	23.90<br>D	0.959<br>D	-	Delete-<br>rious	0.081<br>T	0.985<br>D	-	-	-

EVC2	c.2739G>C<br>(p.Lys913Asn)	rs180747811					conflicting				
NFATC1	c.524G>C<br>(p.Ser175Thr)	rs538981258<br>0.000199681	27.10<br>D	0.988<br>D	-	Benign	0.001<br>D	0.998<br>D	-	RI9<br>Neutral	-
**P4**											
MYH6	c.5410C>A<br>(p.Gln1804Lys)	rs144571463<br>0.00179712	28.90<br>D	0.936<br>D	-	Benign	0.002<br>D	0.996<br>D	-	RI0<br>Disease	-
SEMA3C	c.1009G>A<br>(p.Val337Met)	rs1527482<br>0.0399361	32.00<br>D	0.983<br>D	-	Benign	0.000<br>D	1.000<br>D	-	RI1<br>Neutral	-
**P5**											
ZFPM2	c.2107A>C<br>(p.Met703Leu)	rs121908603<br>0.000798722					Pathogenic				
MESP1	c.156_157insCC<br>GAGCCCCGT<br>(p.Ala53fs)	novel					-				
PCSK9	c.1026A>G<br>(p.Gln342Gln)	rs509504<br>0.981829					conflicting				
**P6**											
NOTCH3	c.4762A>C<br>(p.Asn1588His)	novel	25.90<br>D	0.989<br>D	2.915<br>M	Delete-<br>rious	0.000<br>D	1.000<br>D	-4.650<br>D	RI6<br>Neutral	0.202<br>D
EP300	c.6998C>T<br>(p.Pro2333Leu)	rs750944383	27.80<br>D	0.952<br>D	1.795<br>L	Delete-<br>rious	0.000<br>D	1.000<br>D	-7.560<br>D	RI6<br>Neutral	0.117<br>D
COL6A1	c.2662C>T<br>(p.Arg888Trp)	rs368307185<br>0.000399361					conflicting				
BMP2	c.393A>T<br>(p.Arg131Ser)	rs140417301<br>0.000199681	22.20<br>D	0.670<br>D	3.365<br>M	Delete-<br>rious	0.001<br>D	0.896<br>D	-2.660<br>D	RI0<br>Disease	0.077<br>D
**P7**											
MYH6	c.5410C>A<br>(p.Gln1804Lys)	rs144571463<br>0.00179712	28.80<br>D	0.936<br>D	3.355<br>M	Delete-<br>rious	0.002<br>D	0.996<br>D	-2.430<br>N	RI0<br>Disease	-
FBN1	c.1676C>T<br>(p.Ala559Val)	rs141551765<br>0.000199681	25.70<br>D	0.992<br>D	0.980<br>L	Delete-<br>rious	0.124<br>T	0.960<br>D	-2.940<br>D	RI1<br>Neutral	0.244<br>D
COL6A2	c.185C>T	novel	25.30	0.992	2.398	Delete-	0.000	1.000	-7.430	RI3	0.319

	(p.Pro62Leu)		D	D	M	rious	D	D	D	Neutral	D
**P8**											
DNAH11	c.4306C>T<br>(p.Arg1436Trp)	rs183489539<br>0.00219649	25.20<br>D	0.878<br>D	-	Benign	-	0.819<br>D	-	-	-
MYH6	c.5410C>A<br>(p.Gln1804Lys)	rs144571463<br>0.00179712	28.80<br>D	0.936<br>D	3.355<br>M	Delete-<br>rious	0.002<br>D	0.996<br>D	-2.430<br>N	RI0<br>Disease	-
**P9**											
TTN	c.34216C>A<br>(p.Pro11406Thr)	rs532102837<br>0.000599042					conflicting				
SHROOM3	c.2905C>T<br>(p.Arg969Trp)	rs3733245<br>0.00838658	21.30<br>D	0.568<br>D	1.845<br>L	Delete-<br>rious	0.047<br>D	0.238<br>B	-3.300<br>D	-	-
	c.740A>G<br>(p.Asp247Gly)	rs760622199	25.20<br>D	0.951<br>D	3.290<br>M	Delete-<br>rious	0.002<br>D	0.997<br>D	-6.690<br>D	-	0.036<br>D
**P10**	**Group 2. Mitral valve atresia**										
PCDHB4	c.599A>G<br>(p.Asp200Gly)	novel	25.50<br>D	-	-	-	-	-	-	RI7<br>Disease	-
NOTCH1	c.1699A>G<br>(p.Ile567Val)	rs369067940<br>0.000798722	20.80<br>D	-	-	-	0.019<br>D	0.019<br>B	-	RI9<br>Neutral	-
**P11**											
TTN	c.77836G>A<br>(p.Ala25946Thr)	novel	23.40<br>D	0.951<br>D	-	Delete-<br>rious	0.000<br>D	1.000<br>D	-	-	-
EP300	c.2971G>C<br>(p.Asp991His)	novel	23.70<br>D	0.993<br>D	-	Benign	0.007<br>D	0.145<br>B	-	RI5<br>Disease	-
MYH7	c.1322C>T<br>(p.Thr441Met)	rs121913653					Pathogenic				
**P12**											
TTN	c.73568C>A<br>(p.Pro24523Gln)	rs753557799	23.60<br>D	0.980<br>D	4.460<br>H	Delete-<br>rious	0.012<br>D	-	-5.940<br>D	-	0.104<br>D
CREBBP	c.4490A>C<br>(p.Lys1497Thr)	novel	27.30<br>D	0.988<br>D	2.620<br>M	Delete-<br>rious	0.017<br>D	1.000<br>D	-5.890<br>D	RI6<br>Neutral	0.402<br>D
	c.4485G>C	novel	23.20	0.888	2.620	Delete-	0.027	1.000	-4.910	RI3	0.348

	(p.Lys1495Asn)		D	D	M	rious	D	D	D	Disease	D
**P13**	**Group 3. Double outlet right ventricle**										
LRP2	c.233G>C<br>(p.Gly78Ala)	rs546882372<br>0.000199681	16.48<br>N	0.815<br>D	2.856<br>M	Benign	0.017<br>D	0.985<br>D	-2.930<br>D	RI6<br>Disease	0.266<br>D
NRAP	c.724C>T<br>(p.Pro242Ser)	rs776357401	25.40<br>D	0.992<br>D	2.740<br>M	Delete-<br>rious	0.023<br>D	1.000<br>D	-3.670<br>D	-	0.022<br>T
AXIN1	c.1265G>A<br>(p.Gly422Asp)	rs1187305370	23.50<br>D	0.992<br>D	2.310<br>M	Delete-<br>rious	0.044<br>D	0.998<br>D	-1.490<br>N	RI2<br>Neutral	0.450<br>D
AXIN2	c.1878T>A<br>(p.Ser626Arg)	rs767756290	19.26<br>N	0.672<br>D	2.325<br>M	Delete-<br>rious	0.007<br>D	0.928<br>D	-2.370<br>N	RI8<br>Neutral	0.402<br>D
**P14**											
FOXC1	c.1347_1348insA<br>GC<br>(p.Gly449_Gly45<br>0insSer)	novel					-				
ZFPM2	c.2095C>T<br>(p.His699Tyr)	novel	26.60<br>D	0.967<br>D	-	Delete-<br>rious	0.002<br>D	0.999<br>D	-	RI4<br>Disease	-
**P15**											
TTN	c.92336G>C<br>(p.Arg30779Thr)	novel	23.80<br>D	0.990<br>D	-	Benign	0.184<br>T	0.961<br>D	-	-	-
	c.46847C>T<br>(p.Thr15616Met)	rs368057764	21.50<br>D	0.984<br>D	-	Delete-<br>rious	0.002<br>D	0.971<br>D	-	-	-
NFATC1	c.2251T>G<br>(p.Cys751Gly)	rs754093	18.28<br>N	0.976<br>D	2.765<br>M	Benign	0.010<br>D	-	-5.100<br>D	RI5<br>Neutral	-
NRAP	c.4696C>T<br>(p.Arg1566Cys)	rs1885434	22.30<br>D	0.956<br>D	2.740<br>M	Benign	0.039<br>D	0.014<br>B	-6.520<br>D	**-**	-
SEMA3C	c.1009G>A<br>(p.Val337Met)	rs1527482<br>0.0399361	32.00<br>D	0.983<br>D	-	Benign	0.000<br>D	1.000<br>D	-	RI1<br>Neutral	-
**P16**											
EVC	c.1727G>A<br>(p.Arg576Gln)	rs1383180	24.20<br>D	0.873<br>D	-	Benign	0.124<br>D	1.000<br>D	-	RI7<br>Neutral	-
GDF1	c.985C>T	novel	22.00	0.787	-	Benign	0.000	1.000	-	RI6	-

	(p.Pro329Ser)		D	D			D	D		Neutral	
MYBPC3	c.2504G>T<br>(p.Arg835Leu)	rs527305885<br>0.000399361	27.20<br>D	0.859<br>D	-	Delete-<br>rious	0.002<br>D	0.978<br>D	-	-	-
**P17**											
TBX1	c.928G>A<br>(p.Gly310Ser)	rs41298838					Pathogenic				
MYH7	c.1322C>T<br>(p.Thr441Met)	rs121913653					Pathogenic				
**P18**											
TBX20	c.785C>T<br>(p.Thr262Met)	novel	32.00<br>D	0.994<br>D	-	Delete-<br>rious	0.048<br>D	1.000<br>D	-	RI3<br>Neutral	-
SRCAP	c.6397G>A<br>(p.Val2133Ile)	novel	29.90<br>D	0.980<br>D	-	Benign	0.010<br>D	0.998<br>D	-	RI4<br>Neutral	-
MYH6	c.5410C>A<br>(p.Gln1804Lys)	rs144571463	28.80<br>D	0.936<br>D	3.355<br>M	Delete-<br>rious	0.002<br>D	0.996<br>D	-2.430<br>N	RI0<br>Disease	-
LBX2	c.548C>G<br>(p.Pro183Arg)	rs199798817<br>0.00139776	22.10<br>D	0.635<br>D	-	Benign	0.002<br>D	0.998<br>D	-	RI6<br>Neutral	-
MYOM2	c.656C>T<br>(p.Ala219Val)	rs34823600<br>0.0131789	21.50<br>D	0.771<br>D	-	Benign	0.002<br>D	0.996<br>D	-	RI9<br>Neutral	-
**P19**											
SHROOM3	c.440T>A<br>(p.Leu147His)	rs3821979	22.70<br>D	0.641<br>D	-	Benign	0,001<br>D	0,998<br>D	-	-	-
DNAH6	c.4615C>G<br>(p.Gln1539Glu)	rs571512486<br>0.000599042	25.50<br>D	0.969<br>D	-	Benign	0.000<br>D	0.938<br>D	-	RI4<br>Neutral	-
**P20**											
TTN	c.97733A>T<br>(p.Asn32578Ile)	novel	23.70<br>D	0.988<br>D	4.750<br>H	Delete-<br>rious	0.000<br>D	1.000<br>D	-6.900<br>D	-	0.281<br>D
DNAH11	c.4306C>T<br>(p.Arg1436Trp)	rs183489539<br>0.00219649	25.20<br>D	0.878<br>D	0.895<br>N	Benign	0.001<br>D	-	-3.100<br>D	-	-
NOTCH1	c.7645C>T<br>(p.Arg2549Cys)	rs200893930<br>0.000599042	26.70<br>D	0.988<br>D	0.345<br>N	Delete-<br>rious	0.049<br>D	0.917<br>D	-2.210<br>N	RI9<br>Neutral	0.077<br>D

**P21**											
MESP2	c.306C>A<br>(p.His102Gln)	rs77473319<br>0.00379393	10.70<br>N	0.278<br>N	0.255<br>N	Delete-<br>rious	0.298<br>T	0.014<br>B	-5.500<br>D	RI9<br>Neutral	-
GATA4	c.790G>A<br>(p.Ala264Thr)	rs201520087	25.10<br>D	0.989<br>D	0.870<br>L	Delete-<br>rious	0.048<br>D	-	-1.480<br>N	-	0.061<br>D
DNAH6	c.4218T>G<br>(p.Phe1406Leu)	rs1398626964	22.00<br>D	0.833<br>D	4.500<br>H	Delete-<br>rious	0.000<br>D	1.000<br>D	-5.910<br>D	RI9<br>Neutral	0.151<br>D
COL11A1	c.2227C>A<br>(p.Pro743Thr)	novel	25.40<br>D	0.996<br>D	1.865<br>L	Delete-<br>rious	0.007<br>D	-	-6.330<br>D	-	0.302<br>D
**P22**											
TBX18	c.244_255dupAC<br>GTCTGGGCCG<br>(p.Pro85_Ala86in<br>sThrSerGlyPro)	rs554987935<br>0,00179712					-				
HOXA1	c.215_223delAT<br>CGCCACC<br>(p.His72_His74d<br>el)	rs544314279<br>0,00139776					-				
TTN	c.105876G>A<br>(p.Leu35292Leu)	rs372521529<br>0.000399361					conflicting				
COL3A1	c.3061C>A<br>(p.Leu1021Ile)	rs139619440<br>0.000998403					conflicting				
LRP2	c.6130G>A<br>(p.Ala2044Thr)	rs142266106<br>0.000399361	27.10<br>D	0.966<br>D	1.560<br>L	Delete-<br>rious	0.027<br>D	0.995<br>D	-2.510<br>D	RI4<br>Neutral	0.139<br>D
**P23**											
NKX2-6	c.368G>A<br>(p.Arg123His)	rs568127693<br>0.000399361	17.96<br>N	0.362<br>N	0.895<br>L	Delete-<br>rious	0.357<br>N	-	-0.240<br>N	-	0.267<br>D
MYH11	c.5550G>A<br>(p.Ser1850Ser)	rs146024732<br>0.000199681					conflicting				
NRAP	c.4439A>G<br>(p.Tyr1480Cys)	rs368150420<br>0.000599042	25.50<br>D	0.994<br>D	3.355<br>M	Delete-<br>rious	0.000<br>D	0.996<br>D	-7.860<br>D	-	0.064<br>D
NIPBL	c.7489G>T	rs759115050	18.95	0.988	1.700	Delete-	0.031	0.255	-2.930	RI10	0.184

	(p.Val2497Phe)		N	D	L	rious	D	B	D	Neutral	D
SHROOM3	c.2888C>T<br>(p.Ser963Leu)	rs367782393<br>0.000599042	25.80<br>D	0.808<br>D	2.545<br>M	Delete-<br>rious	0.003<br>D	1.000<br>D	-3.360<br>D	-	-
**P24**											
GATA5	c.374A>T<br>(p.Gln125Leu)	novel	20.60<br>D	0.830<br>D	2.820<br>M	Delete-<br>rious	0.003<br>D	0.979<br>D	-2.850<br>D	RI9<br>Neutral	0.974<br>D
NIPBL	c.3049A>C<br>(p.Ile1017Leu)	rs146714879	21.90<br>D	0.995<br>D	1.735<br>L	Delete-<br>rious	0.014<br>D	0.070<br>B	-0.500<br>N	RI9<br>Neutral	0.136<br>D
SHROOM3	c.2905C>T<br>(p.Arg969Trp)	rs3733245<br>0.00838658	21.30<br>D	0.568<br>D	1.845<br>L	Delete-<br>rious	0.047<br>D	0.238<br>B	-3.300<br>D	-	-
**P25**	**Group 4: Hypoplastic left heart syndrome**										
TTN	c.78855T>C<br>(p.Asp26285Asp)	rs139953862<br>0.00119808					conflicting				
NOTCH1	c.4864C>T<br>(p.Arg1622Cys)	rs747447584	26.90<br>D	0.879<br>D	0.895<br>L	Benign	0.033<br>D	0.989<br>D	-2.120<br>N	RI8<br>Neutral	0.463<br>D
**P26**											
MYH6	c.5410C>A<br>(p.Gln1804Lys)	rs144571463<br>0,00179712	28.80<br>D	0.936<br>D	3.355<br>M	Delete-<br>rious	0.002<br>D	0.996<br>D	-2.430<br>N	RI0<br>Disease	-
CBS	c.598C>T<br>(p.Pro200Ser)	novel	25.50<br>D	0.903<br>D	2.126<br>M	Delete-<br>rious	0.050<br>D	0.978<br>D	-7.110<br>D	RI7<br>Disease	0.622<br>D
**P27**											
TTN	c.105642C>A<br>(p.Phe35214Leu)	rs560557634<br>0,000798722	13.93<br>N	0.692<br>D	0.915<br>L	Delete-<br>rious	0.266<br>N	-	-3.930<br>D	-	0.038<br>D
MYH7	c.2183C>T<br>(p.Ala728Val)	rs121913644<br>0,000199681	25.30<br>D	0.604<br>D	1.255<br>L	Delete-<br>rious	0.011<br>D	0.999<br>D	-2.380<br>N	RI9<br>Disease	0.460<br>D
SRCAP	c.278A>G<br>(p.His93Arg)	rs555507140<br>0,000399361	23.80<br>D	0.992<br>D	1.320<br>L	Delete-<br>rious	0.002<br>D	0.002<br>B	-7.980<br>D	RI6<br>Neutral	0.033<br>D
KDR	c.2524C>T<br>(p.Arg842Cys)	rs41469552	32.00<br>D	0.979<br>D	1.105<br>L	Delete-<br>rious	0.002<br>D		-4.920<br>D		0.222<br>D
**P28**											
MYBPC3	c.1286C>T<br>(p.Ala429Val)	rs370412052						conflicting			

		0,000399361									
TTN	c.75137A>C (p.Lys25046Thr)	rs759445513	22.10 D	0.961 D	1.810 L	Delete-rious	0.007 D	-	-4.140 D	-	0.037 D
MYH6	c.1262T>C (p.Val421Ala)	novel	25.10 D	0.981 D	0.055 L	Delete-rious	0.001 D	0.488 D	-3.180 D	RI8 Disease	0.031 D
**P29**											
TTN	c.10840G>T (p.Glu3614*)	rs540059730 0,000599042					-				
MYH6	c.428G>A (p.Arg143Gln)	rs200377640 0,000199681	32.00 D	0.967 D	1.620 L	Delete-rious	0.001 D	1.000 D	-2.260 N	RI7 Disease	0.094 D

D: Damaging; H: High; L: Low; M: Medium; N: Neutral; P: Polymorphism; T: Tolerant;
